# Distinct Blood and Visceral Adipose Tissue Regulatory T Cell and Innate Lymphocyte Profiles Characterize Obesity and Colorectal Cancer

**DOI:** 10.3389/fimmu.2017.00643

**Published:** 2017-06-09

**Authors:** Gloria Donninelli, Manuela Del Cornò, Marina Pierdominici, Beatrice Scazzocchio, Rosaria Varì, Barbara Varano, Ilenia Pacella, Silvia Piconese, Vincenzo Barnaba, Massimo D’Archivio, Roberta Masella, Lucia Conti, Sandra Gessani

**Affiliations:** ^1^Center for Gender-Specific Medicine, Istituto Superiore di Sanità, Rome, Italy; ^2^Dipartimento di Medicina Interna e Specialità Mediche, Sapienza Università di Roma, Rome, Italy; ^3^Istituto Pasteur Italia-Fondazione Cenci Bolognetti, Rome, Italy

**Keywords:** adipose tissue, fatty acid, obesity, colorectal cancer, immune profile, regulatory T cell, γδ T lymphocyte, NKT-like cell

## Abstract

Visceral adipose tissue (VAT) is a main site where metabolic and immunologic processes interplay to regulate, at local and systemic level, the inflammatory status and immune response. Obesity-associated inflammation and immune dysfunctions are inextricably linked to tumor but, in spite of intense efforts, the mechanisms underpinning this association remain elusive. In this report, we characterized the profile of VAT-associated and circulating innate lymphocyte and regulatory T (T_reg_) cell subsets underlying inflammatory conditions, such as obesity and colorectal cancer (CRC). Analysis of NK, NKT-like, γδ T, and T_reg_ cell populations in VAT and blood of healthy lean subjects revealed that CD56^hi^ NK and OX40^+^ T_reg_ cells are more abundant in VAT with respect to blood. Conversely, CD56^dim^ NK and total T_reg_ cells are most present in the circulation, while γδ T lymphocytes are uniformly distributed in the two compartments. Interestingly, a reduced frequency of circulating activated T_reg_ cells, and a concomitant preferential enrichment of OX40-expressing T_reg_ cells in VAT, were selectively observed in obese (Ob) subjects, and directly correlated with body mass index. Likewise, CRC patients were characterized by a specific enrichment of VAT-associated NKT-like cells. In addition, Ob and CRC-affected individuals shared a significant reduction of the Vγ9Vδ2/γδ T cell ratio at systemic level. The alterations in the relative proportions of T_reg_ and NKT-like cells in VAT were found to correlate with the content of pro- and anti-inflammatory polyunsaturated fatty acids (PUFA), respectively. Overall, these results provide evidence for distinct alterations of the immune cell repertoire in the periphery with respect to the VAT microenvironment that uniquely characterize or are shared by different inflammatory conditions, such as obesity and CRC, and suggest that VAT PUFA composition may represent one of the factors that contribute to shape the immune phenotypes.

## Introduction

Obesity has become a major threat to public health because of its high global prevalence and association with an increased risk of developing chronic diseases. Obesity affects over half a billion adults worldwide, with ~3.5 million attributable deaths each year (1). Similar to gender, race, dietary habits, or smoking history, obesity is one of the risk factors for several types of cancer including colorectal cancer (CRC) (2, 3) and contributes to 3–20% of cancer deaths in western populations (4, 5). CRC is one of the most common gastrointestinal malignant tumors in the world and presents one of the highest rates of morbidity and mortality worldwide (6). Abdominal rather than total adiposity is associated with a 1.5- to 3.5-fold increased risk of developing CRC as compared to lean individuals (7).

Obesity-associated low-grade chronic inflammation is considered a main risk factor for adiposity-related pathologies including CRC (8). Indeed, a well-established link between CRC and chronic inflammation, sustained by tumor cell-extrinsic as well as -intrinsic pathways, has been recognized in multiple settings. It is generally accepted that visceral adipose tissue (VAT)-resident immune cells play a major role in the obesity-associated inflammatory status. Notably, dietary components are recognized as important modulators of inflammation, and healthy/unhealthy diets have been associated with reduced/increased CRC prevalence, respectively (9). Emerging key players in the processes leading to immune surveillance failure that may favor cancer onset in obese (Ob) subjects are the VAT and its composition in fatty acids (FA), in particular the pro-inflammatory polyunsaturated fatty acids (PUFA) (10, 11). Interestingly, dietary intake has been directly linked to PUFA composition of VAT (12) and CRC cell growth as well as tumor progression in mouse models (13). Thus, VAT might represent the initial place where PUFA-related dietary information is transferred to the immune system and contributes to regulate homeostasis.

VAT is immunologically dynamic and contains many different cell types including adipocytes and their progenitors, endothelial cells, and immune cells (14). Recent studies showed clearly that the balance between homeostasis and inflammation in adipose tissue (AT) is mainly controlled by the stromal vascular fraction (SVF) that contains, in homeostatic conditions, a unique repertoire of immune cells (15, 16), whose numbers and activation level have been reported to be altered in obesity. AT homes cells with both pro-inflammatory [M1 macrophages, neutrophils, Th1 CD4^+^ T cells, CD8^+^ T cells, B cells, dendritic cells (DC), and mast cells] and anti-inflammatory [M2 macrophages, regulatory T (T_reg_) cells, Th2 CD4^+^ T cells, eosinophils, and type 2 innate lymphoid cells, ILC2] activity. Among them, T_reg_ cells are a CD4^+^ T cell subset specialized in immune suppression, and in maintaining immune system homeostasis (17). Furthermore, innate lymphocytes have been also recently reported to populate AT (18). In particular, NKT cells, a lineage of T lymphocytes exhibiting NK cell features (19), play beneficial or harmful roles in obesity-associated inflammation and comorbidities (20) as well as in CRC development/progression (21, 22). In addition, NK and γδ T cells are key players in immune surveillance against cancer, and impairment of their function and frequency has been observed in chronic inflammatory conditions (23, 24). Finally, recent studies have highlighted the regulatory role of ILC2 in AT metabolism and homeostasis (16). Thus, AT homeostasis is maintained through the regulation of the immune cell profile, and obesity skews this balance toward a pro-inflammatory status. The tumor-promoting effects of obesity occur not only at local level, *via* altered VAT microenvironment, but also systemically, *via* dysregulated immune cell profile and circulating inflammatory factors that mirror adipose inflammation. However, the alterations in immune cell repertoires occurring in the peripheral blood (PB), VAT, and proximal tissues deserve further investigation in order to elucidate the extent of immune dysregulation in obesity that may set the basis for cancer development.

In this study, we investigated the profile of human VAT-associated and systemic γδ T, NK, NKT-like, and T_reg_ cells in lean and obese (Ob) subjects, affected or not by CRC. We report that in healthy lean subjects innate lymphocyte subsets and T_reg_ cells exhibit a differential distribution in blood with respect to VAT. Furthermore, we identify alterations of the immune cell profile specific for Ob subjects, such as a reduced level of circulating activated T_reg_ (aT_reg_) cells paralleling a preferential enrichment of OX40-expressing T_reg_ cells in VAT, or for CRC patients, such as an increased VAT-associated NKT-like cell frequency. In addition, obesity and CRC share a significant reduction of the Vγ9Vδ2/γδ T cell ratio at systemic level. Of note, the alterations in the relative proportions of T_reg_ and NKT-like cells in VAT correlate with the its content of pro- and anti-inflammatory PUFA, respectively, in both pathological conditions.

Overall, these results provide evidence for distinct alterations of the immune cell repertoire in the periphery with respect to the VAT microenvironment that uniquely characterize, or are shared by, obesity and CRC, and suggest a role for VAT PUFA composition in shaping immune phenotypes.

## Materials and Methods

### Patients and Samples

Human VAT biopsies and blood samples from the same individual were collected from lean and Ob subjects undergoing abdominal surgery or laparoscopy for benign (i.e., gallbladder disease without icterus, umbilical hernia, and uterine fibromatosis) or CRC conditions (histologically proved primary colon adenocarcinoma, stage TNM 0–III). The exclusion criteria were as follows: clinical evidence of active infection, recent (within 14 days) use of antibiotics/anti-inflammatory drugs, pregnancy, hormonal therapies, severe mental illness, autoimmune diseases, family history of cancer, other neoplastic diseases. Subjects belonging to four groups were enrolled: normal weight (Nw), Ob, Nw with CRC (Nw/CC), and Ob with CRC (Ob/CC). In the Nw groups, the body mass index (BMI) range was 18–24.9 kg/m^2^. In the Ob groups, BMI was ≥30 kg/m^2^, and waist circumference ≥95 cm for men and ≥80 cm for women. For each category, the number of subjects ranged from a minimum of 6 to 16 for Nw, 4 to 15 for Ob, 6 to 13 for Nw/CC, 6 to 10 for Ob/CC. The different quantity of biological samples available for each single donor did not allow to perform all the analyses on the same number of subjects.

Blood samples were drawn at the time of obtaining peripheral vein access for surgery. Peripheral blood mononuclear cells were separated by Ficoll-Hypaque density-gradient centrifugation and collected in complete RPMI 1640 medium containing 10% FBS, 2 mM l-glutamine, penicillin/streptomycin (Euroclone). VAT biopsies were microdissected, rinsed several times in 0.9% NaCl, and digested with 5 ml of Krebs-Ringer solution (0.12 M NaCl, 4.7 M KCl, 2.5 mM CaCl_2_, 1.2 mM MgSO_4_, 1.2 mM KH_2_PO_4_) containing 20 mM HEPES pH 7.4, 3.5% fatty acid-free BSA, 200 nM adenosine, 2 mM glucose, and type 1 collagenase for 1 h (1 mg/g tissue) at 37°C in shaking water bath. VAT SVF were obtained as previously described (25). Briefly, 15–40 g of VAT biopsies were microdissected and extensively washed with sterile PBS to remove contaminating erythrocytes. The extracellular matrix was digested with 0.1% type I collagenase at 37°C, and shaken vigorously for 60 min in shaking water bath to separate the stromal cells from primary adipocytes. Dissociated tissue was filtered to remove debris, and centrifuged at 1,500 rpm for 10 min. The suspending portion containing lipid droplets was discarded and the cell pellet was resuspended, washed twice, and cultured in complete RPMI 1640.

### Flow Cytometry

Peripheral blood mononuclear cells and VAT SVF cells were stained with fluorochrome-labeled antibodies CD3 (FITC), CD4 (FITC or BrilliantViolet 785), CD45RA (APC or BrilliantViolet 605), CD127 (PeCy7), γδ TCR (PE), Vδ2 (FITC), CD56 (BrillantViolet 421), OX40 (PE), CD16 (APC). Intracellular staining for FOXP3 was performed using the anti-FOXP3 (PE or PerCP-Cy5.5) mAb and FOXP3 Transcription Factor Staining Buffer Set according to manufacturer’s instructions (eBioscience). At first, cells were incubated 30 min at room temperature (RT) with Fixable Viability Dye (eFluor 780, eBioscience) to stain and exclude dead cells, then staining with antibodies for surface antigens was performed for 20 min at 4°C. FOXP3 intracellular staining was performed incubating cells for 30 min at RT. Data were acquired on LSR Fortessa (Becton Dickinson) and analyzed with FlowJo software (Tree Star Inc., version 10.1r5) or on FACSCalibur flow Cytometer (BD Biosciences) and analyzed with the Cell Quest Pro software.

### FA Analysis

Total lipids from VAT samples were extracted with chloroform–methanol 2:1 (v/v) and FA methyl esters prepared (26) and analyzed as previously described (10). The individual FA detected were expressed as a percent of total FA.

### Statistical Analysis

GraphPad Prism 5 software was used for statistical analysis. Statistical comparison between groups was performed by the one-way analysis of variance with Newman–Keuls *post hoc* test and by the two-tailed unpaired Student’s *t*-test for independent samples, as appropriate. Comparisons were expressed as means from several experiments ± SEM. Pearson’s test was performed to determine simple correlations between two variables. Differences were considered significant when *p* values were <0.05.

## Results

### Human Innate Lymphocytes and T_reg_ Cells Are Differentially Distributed in PB and VAT in Healthy Subjects

**Figure 1:**
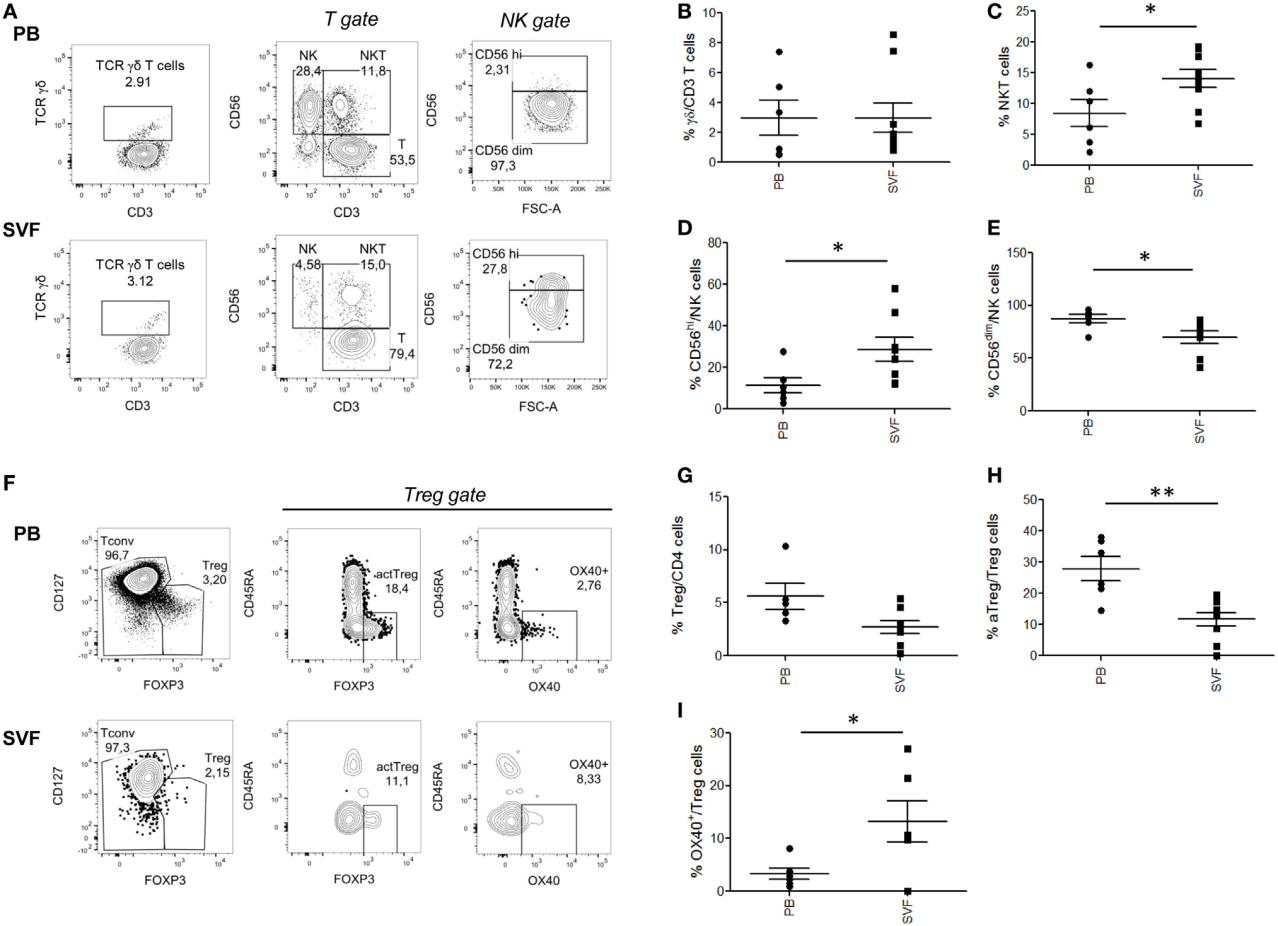
Composition of major T lymphocyte subpopulations in visceral adipose tissue (VAT) stromal vascular fraction (SVF) versus peripheral blood (PB) of lean healthy subjects. Cell subset percentages within total PB and VAT SVF lymphocytes of nine healthy lean subjects were analyzed. Gating strategies for NK, NKT, T, and γδ T cells **(A)**, total regulatory T (T_reg_), conventional T cells (T_conv_), OX40^+^ T_reg_ and activated T_reg_ cells (aT_reg_) **(F)**, in PB and SVF from a representative donor. γδ T cells, TCRγδ^+^CD3^+^ **(B)**; NK cells, CD3^−^CD56^+^ **(D,E)**; NKT cells, CD3^+^CD56^+^ **(C)**; T_reg_ cells, CD4^+^FOXP3^+^CD127^low^ **(G)**; aT_reg_, FOXP3^high^CD45RA^−^ **(H)**; OX40^+^ T_reg_ cells **(I)**. Each dot represents an individual donor. Mean ± SEM is shown for each group. *p < 0.05, **p < 0.01, by unpaired Student’s *t*-test.

Several immune cell subsets populate the AT, and alterations of their relative numbers and functions have been suggested to influence obesity-associated local and systemic inflammation as well as CRC development/progression. We initially quantified the frequency of innate lymphocyte populations (γδ T, NK, NKT-like cells) and T_reg_ cells in homeostatic conditions assessing matched VAT SVF and PB samples collected from healthy lean subjects. The gating strategies and representative plots for each cell population are reported in Figures 1A,F and Figure S1 in Supplementary Material. As shown in Figure 1, no significant differences were observed in the tissue distribution of total γδ T lymphocytes (Figure 1B). However, the analysis of other innate cell populations revealed a preferential accumulation of the CD56^hi^ NK cell subset in VAT SVF as compared to PB (Figure 1D), whereas the majority of circulating NK cells was CD56^dim^ (Figure 1E). In keeping with previously published data highlighting that NKT cells are one of the AT resident cell subsets, a general enrichment of the CD3^+^CD56^+^ NKT-like cell population was found in VAT SVF (Figure 1C) as compared to PB. Concomitant with the accumulation of NKT-like cells, a significant decrease of T_reg_ cell (CD4^+^FOXP3^+^CD127^low^) frequency was observed at local level as compared to PB (Figure 1G). Recently, different T_reg_ cell subpopulations have been described, such as aT_reg_ cells which have suppressive function, resting T_reg_ (rT_reg_) cells which can convert to aT_reg_, and non-suppressive T_reg_ (nsT_reg_) cells (27). Moreover, the OX40-expressing T_reg_, cell subset, with strong suppressive activity and high proliferative potential, has been described to be associated with tissue localization and especially cancer microenvironments (28, 29). Of note, when these cell subsets were characterized, an opposite tissue distribution was observed between aT_reg_ and OX40-expressing T_reg_ cells (Figures 1H,I). Specifically, a significantly higher frequency of aT_reg_ cells was detected at systemic with respect to local level (Figure 1H). Conversely, OX40-expressing cells were found selectively distributed in VAT SVF (Figure 1I), in line with the role of OX40 in imparting preferential survival to T_reg_ cells in tissues.

### Obesity Is Associated with a Reduced Frequency of Circulating aT_reg_ Cells and a Preferential Enrichment of OX40-Expressing T_reg_ Cells in VAT

**Figure 2:**
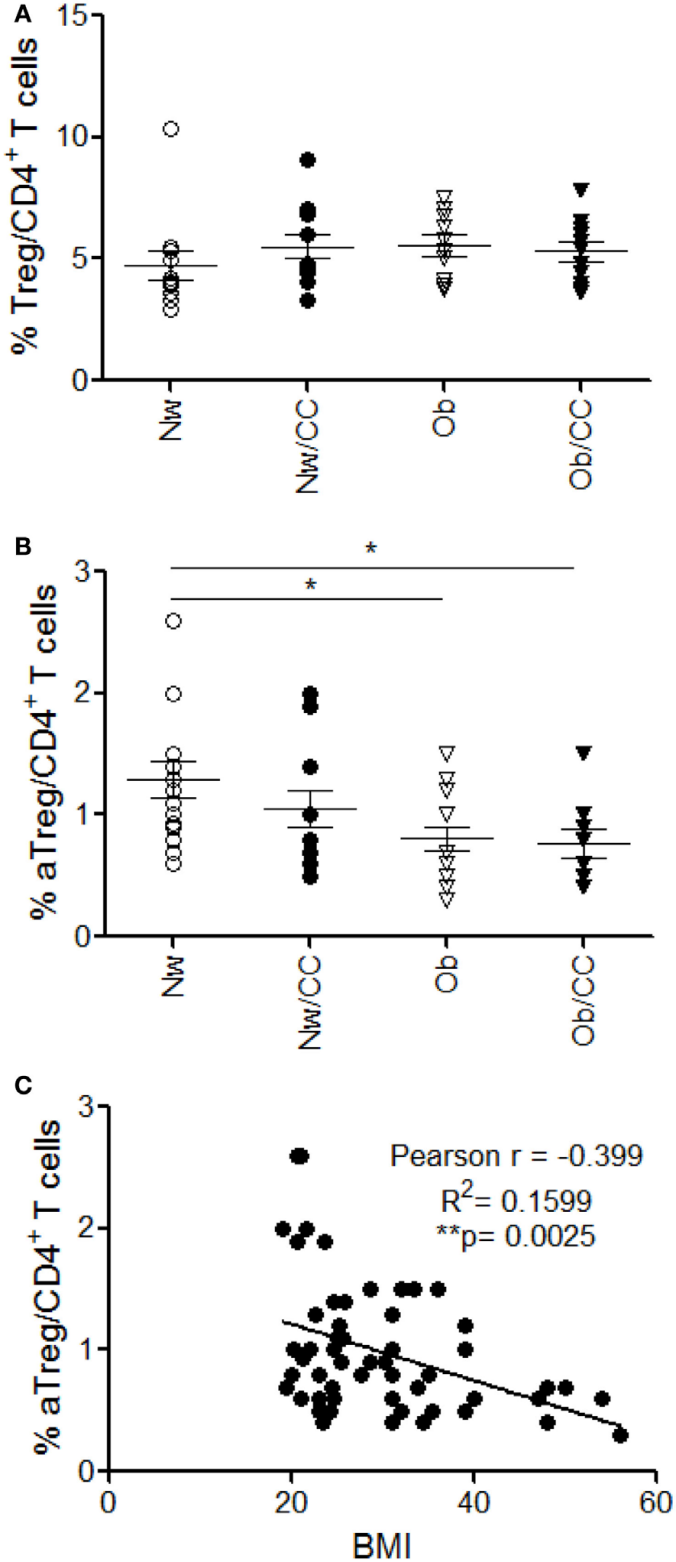
A significant reduction of circulating CD45RA^−^FOXP3^hi^ activated T_reg_ (aT_reg_) cells occurs in obese (Ob) subjects: correlation with body mass index (BMI). Peripheral blood (PB) lymphocytes isolated from lean normal weight (Nw), Ob, lean affected by colorectal cancer (CRC) (Nw/CC), and Ob affected by CRC (Ob/CC) donors were analyzed by flow cytometry. Frequency of regulatory T (T_reg_) (CD4^+^FOXP3^+^CD127^low^) cells **(A)** and aT_reg_ cells (CD4^+^FOXP3^high^ CD45RA^−^) **(B)** was estimated in PB of the four groups. Each dot represents an individual donor. Mean ± SEM is shown for each group. *p < 0.05 by analysis of variance. **(C)** Pearson’s correlation (*r*) between aT_reg_ cell frequency and BMI in all subjects (**p < 0.01).

In chronic inflammatory diseases, such as obesity and cancer, the effector/regulatory equilibrium may be persistently perturbed, finally contributing to tumor progression by suppressing antitumor immunity (30). Therefore, we compared the frequency of T_reg_ cells in PB of lean and Ob subjects, affected or not by CRC. Anthropometric and clinical parameters of study subjects are shown in Table 1. Although no changes in the frequency of total T_reg_ cells were observed among the four donor categories studied (Figure 2A), a deeper analysis of T_reg_ cell subsets revealed a significantly reduced frequency of circulating aT_reg_ cells in Ob individuals, independent of CRC (Figure 2B). Notably, correlation analysis showed that the frequency of this subpopulation inversely correlates with BMI (Figure 2C). The percentage of rT_reg_ and nsT_reg_ cell subsets was not affected by obesity or CRC (Figure S2 in Supplementary Material).

**Table 1:** Anthropometric and clinical parameters of study subjects.

Subjects	Nw	Ob	Nw/CC	Ob/CC
Number (54)	16	15	13	10
Body mass index (kg/m^2^)	23.9 ± 0.78	41.22 ± 8.58	22.50 ± 1.93	32.79 ± 2.17
Age (years)	46.07 ± 12.83	32.83 ± 11.02	61.08 ± 12.85	65 ± 10.3
Sex (female/male)	8/8	7/8	9/4	3/7
Stage 0	–	–	1	1
Stage I	–	–	5	3
Stage II	–	–	3	3
Stage III	–	–	4	3

**Figure 3:**
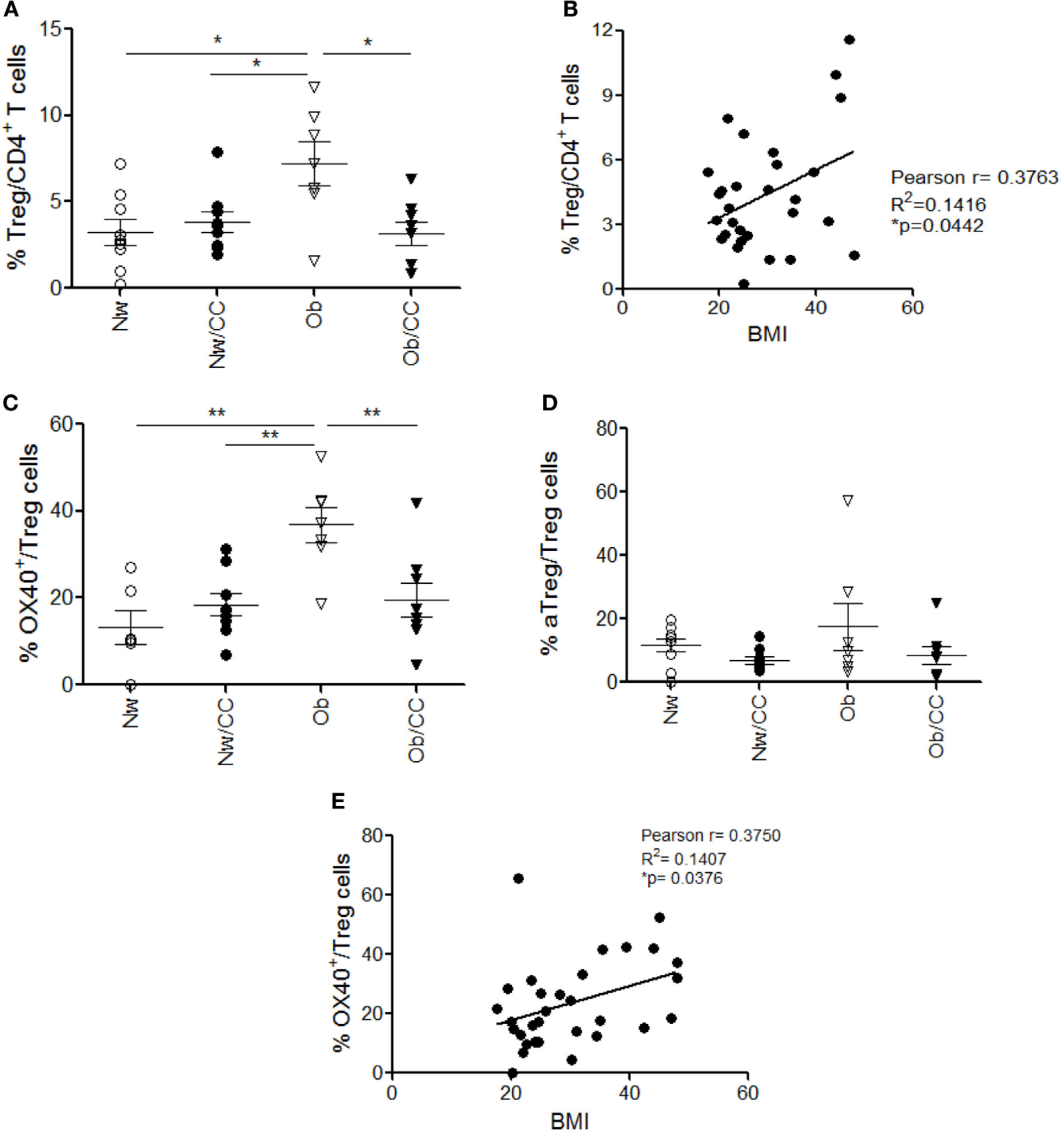
A significant increase of total regulatory T (T_reg_) cells and a preferential enrichment of OX40^+^ T_reg_ cells occur in visceral adipose tissue (VAT) stromal vascular fraction (SVF) from obese (Ob) subjects: correlation with body mass index (BMI). VAT SVF lymphocytes isolated from lean normal weight (Nw), Ob, lean affected by colorectal cancer (CRC) (Nw/CC), and Ob affected by CRC (Ob/CC) donors were analyzed by flow cytometry. Frequencies of T_reg_ (CD4^+^FOXP3^+^CD127^low^) cells **(A)**, OX40^+^ T_reg_ cells **(C)**, and activated T_reg_ cells (CD4^+^FOXP3^high^CD45RA^−^) **(D)** were estimated in VAT SVF of the four donor groups. Each dot represents an individual donor. Mean ± SEM is shown for each group. *p < 0.05, **p < 0.01 by analysis of variance. Pearson’s correlation (*r*) between T_reg_ **(B)** or OX40^+^ T_reg_ **(E)** cell frequencies and BMI, in all subjects (*p < 0.05).

The analysis of T_reg_ cells at the tissue level revealed that these cells accumulate in VAT SVF of Ob individuals, but not of CRC-affected subjects (Figure 3A), and that their frequency positively correlates with BMI (Figure 3B). Based on the pivotal role of OX40 in supporting T_reg_ cell fitness (28, 31) and on its selective expression in VAT-associated T_reg_ cells in healthy lean subjects (Figure 1I), we investigated whether the accumulation of this cell population observed in VAT SVF of Ob individuals was associated with a higher expression of OX40. Indeed, we found that the frequency of OX40-expressing T_reg_ cells was significantly higher in Ob subjects with respect to all other categories of individuals (Figure 3C). Conversely, the frequency of aT_reg_ cells remained unchanged (Figure 3D). Interestingly, the extent of OX40 expression in T_reg_ cells showed a positive correlation with BMI (Figure 3E). As shown in Figure S3 in Supplementary Material, an increased expression of OX40 was also found in VAT-associated CD4^+^FOXP3^−^ conventional T cells (T_conv_) from Ob subjects (Figure S3A in Supplementary Material); however, the frequency of OX40^+^ T_conv_ cells did not correlate with BMI (Figure S3B in Supplementary Material). These data suggest that, in the VAT microenvironment, obesity prompts T_reg_ cell expansion, possibly as a negative feedback mechanism to counteract inflammation. Notably, this event seems disrupted in VAT of CRC patients, despite obesity.

### A Selective Enrichment of VAT-Associated NKT-Like Cells Characterizes CRC Condition

**Figure 4:**
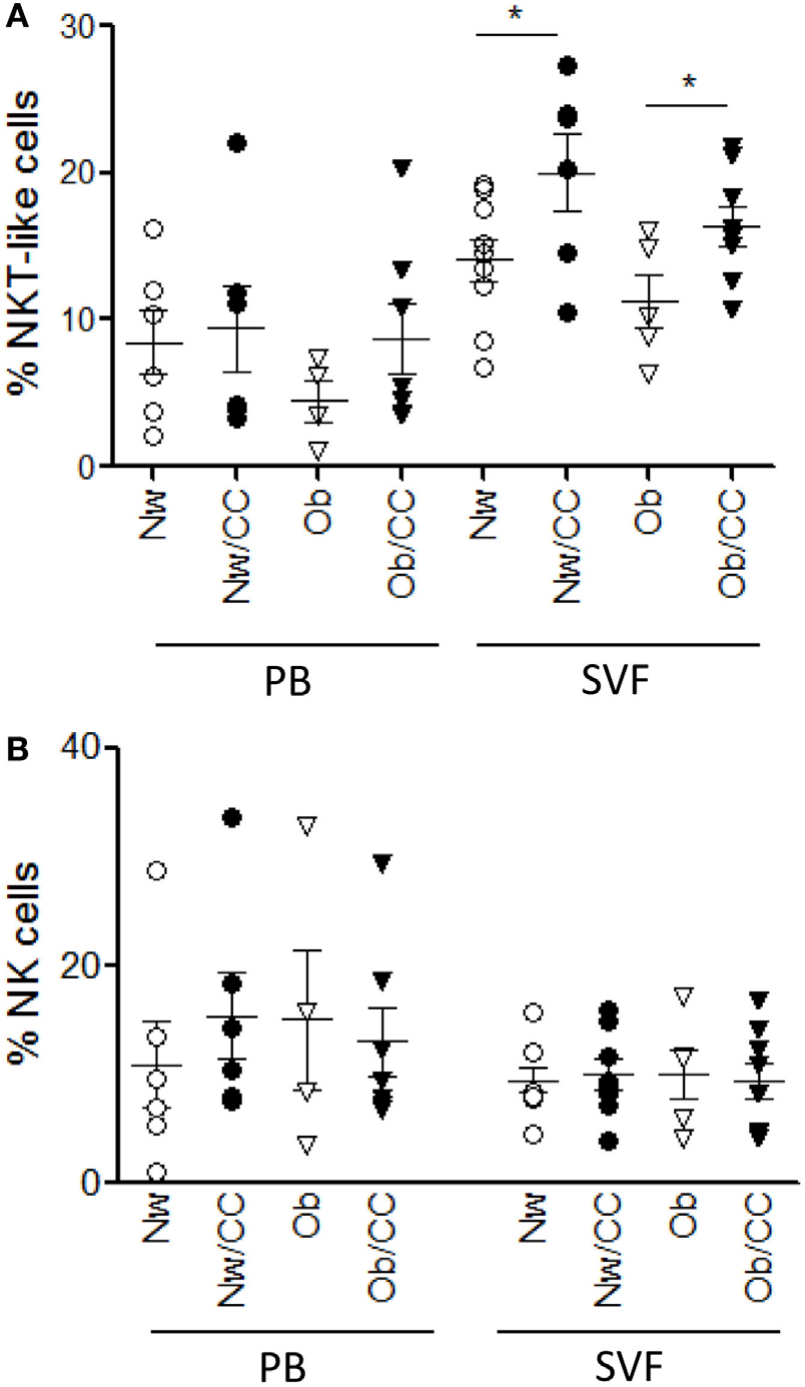
The frequency of visceral adipose tissue (VAT)-associated NKT cells, but not NK cells, is increased in colorectal cancer (CRC)-affected subjects as compared to cancer-free donors. VAT stromal vascular fraction (SVF) and peripheral blood (PB) lymphocytes isolated from lean normal weight (Nw), obese (Ob), lean affected by CRC (Nw/CC), and Ob affected by CRC (Ob/CC) donors were analyzed by flow cytometry. Frequencies of NKT (CD3^+^CD56^+^) **(A)** and NK (CD3^−^CD56^+^) **(B)** cells were evaluated in the four categories of donors. Each dot represents an individual donor. Mean ± SEM is shown for each group. *p < 0.05, by analysis of variance.

To investigate whether the frequency of NK and NKT-like cells at systemic and VAT level could be affected by obesity and/or CRC, flow cytometric analysis of these subsets was also performed in the four categories of subjects. As shown in Figure 4A, CRC condition, rather than obesity, is characterized by an enrichment of CD3^+^CD56^+^ NKT-like cells. Specifically, a higher frequency of this cell population was detected in VAT SVF from CRC-affected subjects as compared to cancer-free individuals, irrespective of their BMI. Conversely, no changes in the frequency of total NK cells were observed in VAT SVF and PB among all categories of subjects (Figure 4B). Moreover, the tissue distribution of the CD56^hi^ and CD56^low^ NK cell subsets was not affected by obesity or CRC (data not shown).

### Obesity and CRC Share a Reduced Vγ9Vδ2/γδ T Cell Ratio at Systemic Level

**Figure 5:**
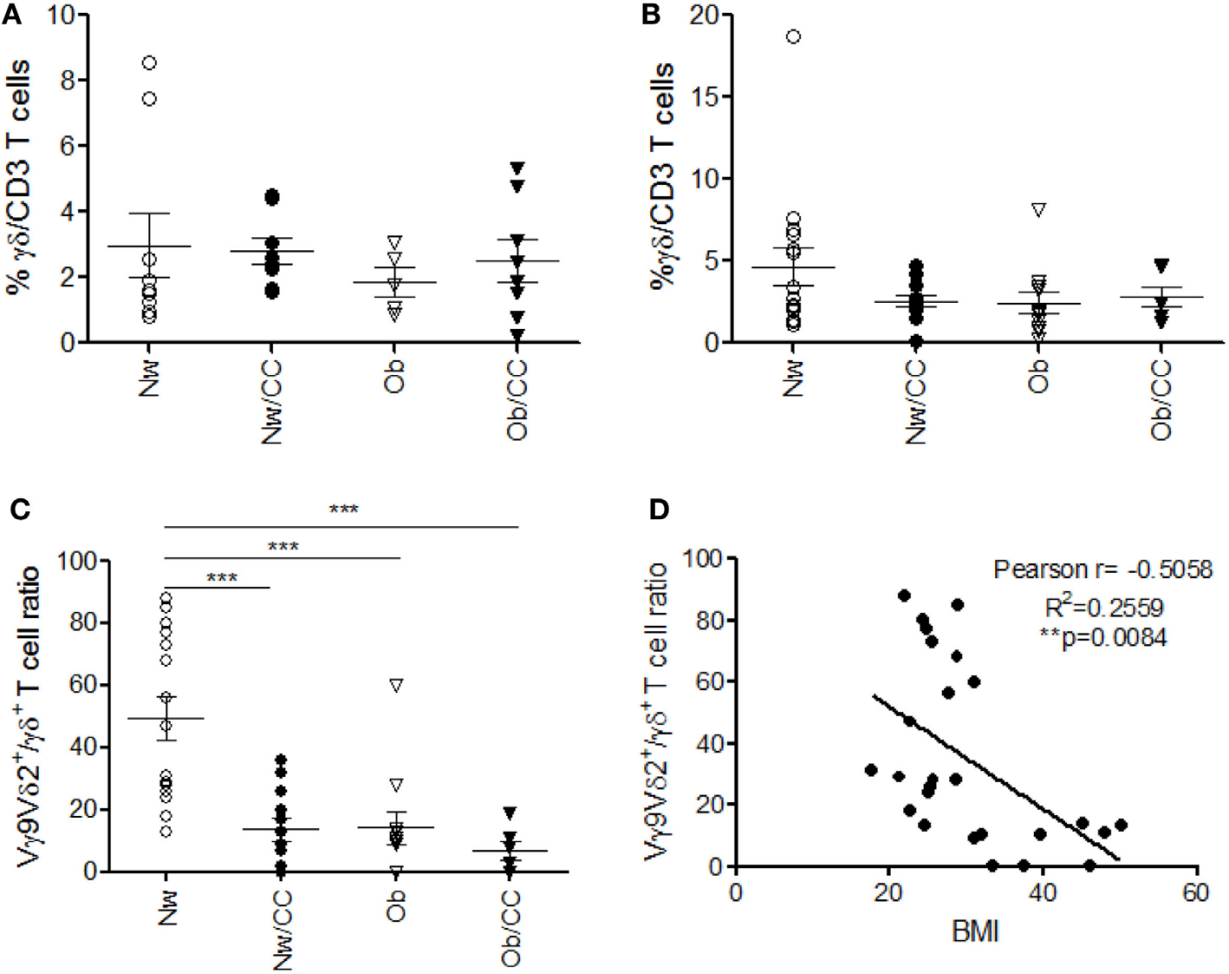
Obese (Ob) and colorectal cancer (CRC)-affected individuals exhibit normal frequencies of peripheral blood (PB) and visceral adipose tissue (VAT)-associated total γδ T lymphocytes but a significantly reduced Vγ9Vδ2/γδ T cell ratio in PB that correlates with body mass index (BMI). VAT stromal vascular fraction (SVF) and PB lymphocytes isolated from lean normal weight (Nw), Ob, lean affected by CRC (Nw/CC), and Ob affected by CRC (Ob/CC) donors were analyzed by flow cytometry. Frequency of total γδ T cells (among CD3^+^ T cells) was evaluated in SVF **(A)** and PB **(B)**. **(C)** Vγ9Vδ2/γδ T cell ratios (PB) were calculated in the four categories of donors. Each dot represents an individual donor. Mean ± SEM is shown for each group. ***p < 0.001, by analysis of variance. **(D)** Pearson’s correlation (*r*) between the Vγ9Vδ2/γδ T cell ratio and BMI in Nw and Ob subjects (**p < 0.01).

Among γδ T lymphocytes, the main circulating subset Vγ9Vδ2 has been involved in immunosurveillance against cancer, and impairment of its function and frequency has been observed in chronic inflammatory conditions (24). We thus investigated the distribution of γδ T cells and of the Vγ9Vδ2 subset, in PB with respect to VAT, in the four categories of subjects. Although no significant differences were observed in the frequency of VAT-associated (Figure 5A) and PB (Figure 5B) total γδ T lymphocytes (Figure 5), when the analysis was extended to the Vγ9Vδ2 subset, a significant reduction of the Vγ9Vδ2/γδ T cell ratio was found in the systemic compartment of Ob and CRC-affected subjects as compared to healthy lean individuals (Figure 5C). Of note, an inverse correlation was observed between the Vγ9Vδ2/γδ T cell ratio and BMI when Ob and lean subjects were considered (Figure 5D).

### T_reg_ and NKT-Like Cells Are Inversely Distributed in VAT and Their Frequencies Correlate with Individual PUFA Content

**Figure 6:**
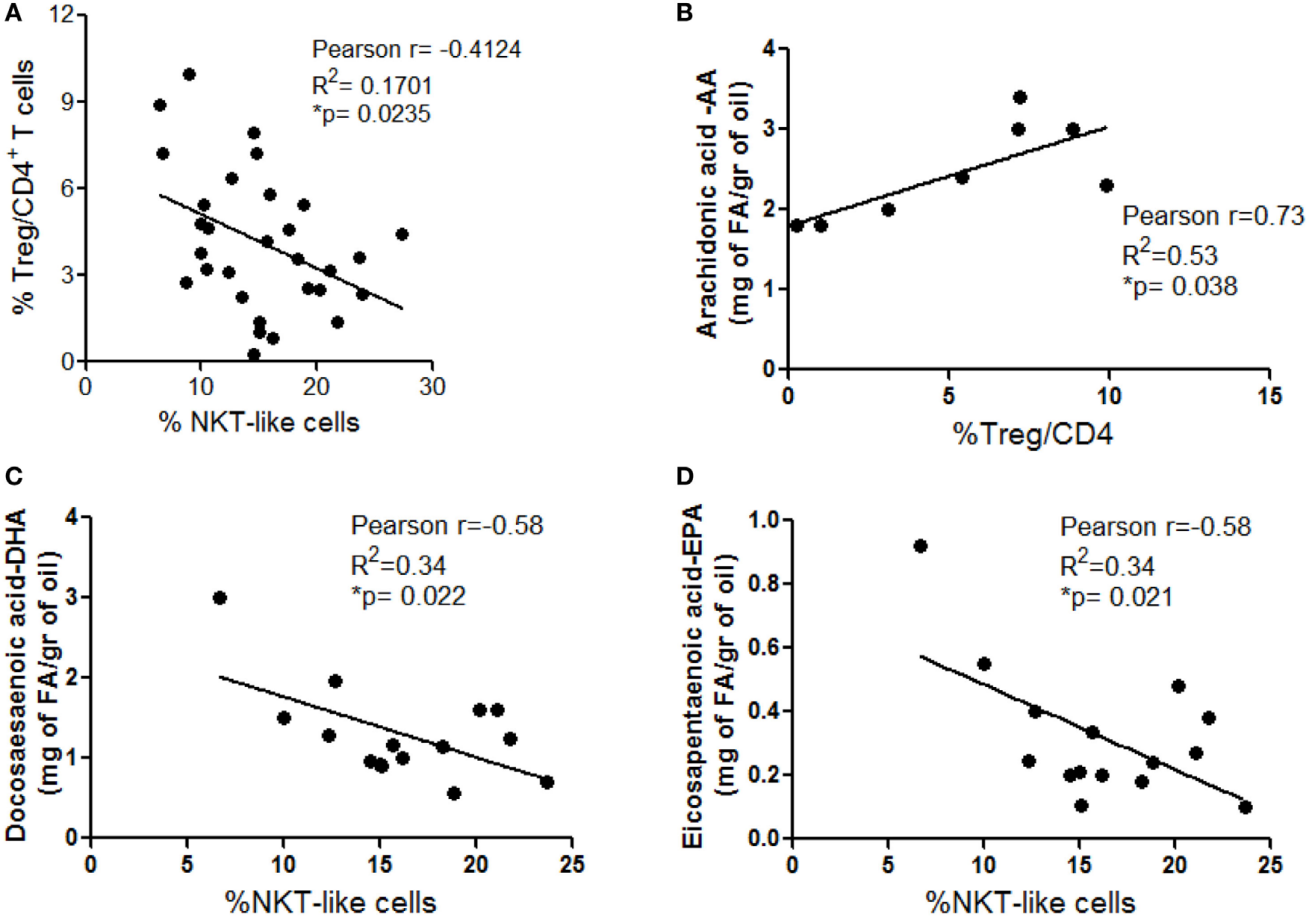
Inverse distribution between total visceral adipose tissue (VAT) stromal vascular fraction (SVF) regulatory T (T_reg_) and NKT-like cells and correlation with VAT polyunsaturated fatty acid content. Pearson’s correlation between: total T_reg_ and NKT-like cell frequencies in VAT SVF **(A)**, arachidonic acid (AA) content, evaluated in visceral adipocytes, and T_reg_ cell frequency estimated in VAT SVF isolated from lean normal weight (Nw) and obese (Ob) donors **(B)**, and docosahexaenoic (DHA) **(C)** and eicosapentaenoic acid (EPA) **(D)** content, evaluated in visceral adipocytes, and NKT cell frequency estimated in VAT SVF isolated from lean (Nw), lean affected by colorectal cancer (CRC) (Nw/CC) and Ob affected by CRC (Ob/CC) donors.

An interplay between NKT and T_reg_ cells has been described in different settings. In particular, NKT cells can promote T_reg_ cell induction and, conversely, T_reg_ cells can exhibit suppressive activity on NKT cells (32, 33). Based on this knowledge and on our observation that these immune cell subsets are differently distributed in VAT SVF from Ob and CRC subjects (Figures 3A and 4A), a correlation analysis between the frequencies of total T_reg_ and NKT-like cells was performed to assess the influence of obesity and CRC conditions on this relationship in human VAT. Interestingly, as shown in Figure 6A, we observed a significant inverse correlation between these cell populations, indicative of a potential negative interplay. This inverse relation may explain the alternate prevalence of NKT-like cells in VAT of CRC-affected patients, and of T_reg_ cells in VAT of CRC-free Ob subjects (Figures 3A and 4A).

We have previously demonstrated a significant decrease in the ω3/ω6 PUFA ratio in Ob and CRC-affected subjects with respect to healthy lean individuals (10). Therefore, we investigated whether obesity- or cancer-associated modulation of specific immune cell subsets could be somehow related to distinct PUFA profiles, focusing on individual ω3 and ω6 PUFA. Correlation analysis showed that the T_reg_ cell frequency in VAT SVF positively correlates with ω6 PUFA arachidonic acid (AA) content when lean and Ob subjects were considered (Figure 6B). Likewise, the frequency of VAT-associated NKT-like cells in healthy lean and CRC subjects inversely correlated with the content of two key anti-inflammatory ω3 PUFA, docosahexaenoic (DHA) and eicosapentaenoic acid (EPA) (Figures 6C,D). These results suggest that PUFA composition may represent one of the factors that influence VAT microenvironment.

## Discussion

In this study, we performed a comparative analysis of circulating versus VAT-associated immune cell subsets unraveling not only a different distribution of specific subpopulations in PB versus VAT but also alterations in one or in another compartment that are associated with obesity or CRC condition. Specifically we observed that the frequency of circulating aT_reg_ cell subset is reduced in Ob individuals, irrespective of CRC, and inversely correlates with BMI, likely reflecting the inflammatory status. In this respect, a previous study reported that circulating CD4^+^CD25^+^FOXP3^+^ T_reg_ cells are reduced in Ob individuals and their frequency inversely correlates with biomarkers of inflammation, weight, BMI, and leptin levels (34). We did not observe a significant reduction in total T_reg_ cell frequency. However, a deeper characterization of T_reg_ cell subsets revealed, for the first time, a selective reduction of circulating aT_reg_ cells in obesity. In addition, this is the first study in which a comparative analysis between circulating and VAT-associated T_reg_ cells has been carried out in lean and Ob individuals. We found that T_reg_ cells accumulate in VAT of Ob, but not lean, subjects and this was likely due to a protective response to the inflammatory status typical of Ob subjects. In keeping with this hypothesis, we found a positive correlation between the extent of adiposity defined by BMI and the frequency of a VAT-associated T_reg_ cell population, highly expressing OX40 (OX40^+^ T_reg_), a marker associated with T_reg_ cell survival and suppressive function (28). Therefore, apparently, the VAT microenvironment characterizing obesity reproduces a condition that is favorable to T_reg_ cell accumulation, in particular of the OX40^+^ subset, probably as a result of the need to counteract tissue inflammation. Contrasting results have been obtained in mouse and human models concerning the role of T_reg_ cells in regulating the inflammatory status of AT. While a clear-cut decrease of T_reg_ cells was detected in the VAT of Ob mice (35–37), inversely correlating with macrophage infiltration (36), a rather different situation was observed in humans. In keeping with our results, Zeyda and coworkers reported that systemic and AT inflammation positively correlate with T_reg_ cell abundance within the Ob group, as assessed by FOXP3 transcript expression (38). Likewise, Pereira and colleagues found a greater FOXP3 mRNA expression in VAT of Ob individuals that positively correlated with IL-6 and TNFα expression as well as BMI (39). In our study, a deeper phenotypic characterization of VAT-associated T_reg_ cells has been performed, highlighting, for the first time, the role of OX40 in driving their enrichment in VAT.

In this regard, we have previously demonstrated that OX40^+^ T_reg_ cells, expanded in cirrhotic liver, hepatocellular carcinoma, and CRC, exhibit a phenotype (Helios^hi^, CD39^hi^) compatible with a strong suppressive activity, a high proliferative potential and a stable regulatory program, suggesting that CRC may take advantage from creating a favorable milieu for their accumulation (29). In this study, the lack of accumulation of T_reg_ cells in VAT of CRC-affected Ob subjects might result from their recruitment at the tumor site, an advantageous event facilitating tumor progression. Unfortunately, matched tumor tissues were not available at the time of analysis to conclusively ascertain the frequency and phenotype of T_reg_ cells at the tumor site. The role of VAT-associated T_reg_ cells in CRC has been only poorly investigated. Conversely, a number of studies have reported increased blood and tumor tissue infiltrating T_reg_ cell numbers in CRC patients (40). In particular, an increase in aT_reg_ cell number has been associated with advanced/metastatic tumor stage (41). Our failure in detecting circulating aT_reg_ cell increase in CRC-affected subjects might be explained by the fact that they have been analyzed at earlier stages of disease. Collectively, our results delineate a network of diverse T_reg_ cell subsets, differently located in blood and VAT, that contribute to obesity-associated immune dysfunctions.

NKT cells have been shown to play beneficial or harmful roles in obesity-associated inflammation and co-morbidities (20), as well as in CRC development/progression (21, 22). Accumulation of total CD3^+^CD56^+^ NKT-like cells has been reported in human VAT with a decrease of the invariant NKT cell subset associated with severe obesity and CRC (42). In keeping with this study, we found a selective enrichment of CD3^+^CD56^+^ NKT-like cells in VAT as compared to blood in healthy individuals and reported new evidence for their preferential accumulation in VAT of CRC patients, independent of BMI. Conversely, no major changes were observed in the periphery. Interestingly, we observed an opposite distribution of T_reg_ and NKT-like cells, both endowed with immunoregulatory/suppressor activity, in VAT from Ob and CRC subjects. Accordingly, when all categories of subjects were analyzed, the frequency of NKT-like cells inversely correlated with that of T_reg_ cells. In this regard, a reciprocal regulation between these two immune cell populations has been reported in other experimental settings (32, 33), suggesting two different scenarios: (i) a bidirectional regulation of their frequency or (ii) redundant function but different localization depending on the pathophysiological condition. Overall, we could envisage the following scenario at the level of VAT: obesity results in enrichment of T_reg_ cells as a mechanism to attenuate excessive inflammation. However, their presence could generate an immunosuppressive environment favoring tumor establishment. *Vice versa*, once the tumor has established, NKT-like cells might be recruited to the VAT. In this regard, the frequency of NKT-like cells at the tumor site has been positively correlated with significantly longer overall and disease-free survival rates, although the effector mechanisms were not identified (43).

In addition to alterations selectively associated with obesity or CRC, we demonstrate that specific innate cell subsets are affected in both pathologies. We provide herein the first evidence for a reduced Vγ9Vδ2/γδ T cell ratio in the PB of CRC-affected individuals. Moreover, such a reduction was detected in Ob subjects with respect to lean individuals, as recently reported (44) and the Vγ9Vδ2/γδ T cell ratio was found to inversely correlate with BMI. Owing to the protective role of Vγ9Vδ2 T cell-mediated responses in tumor surveillance, obesity- and CRC-induced reduction in this cell subset might be detrimental for the control of tumor development and growth. Interestingly, due to their capacity to specifically recognize and kill CRC initiating cells, γδ T lymphocytes have been proposed as a promising tool for novel immunotherapeutic strategies in patients with CRC (45). As obesity represents a risk factor for CRC and both inflammatory conditions are characterized by loss of Vγ9Vδ2 cells, we speculate that the impairment of this subset in obesity might contribute to CRC immune escape and development.

We have previously demonstrated that specific inflammatory signatures characterize the VAT of Ob and CRC subjects (10). In particular, a reduced ω3/ω6 PUFA ratio and alterations of individual ω6 PUFA profile have been observed in these subjects (10, 11). Among the different factors potentially influencing VAT microenvironment and immune cell distribution, the relative ω3/ω6 PUFA composition, which reflects their dietary intake, might play a pivotal role by virtue of the capacity of these molecules to exert anti- or pro-inflammatory activities, respectively, and to influence immune cell functions (46, 47). We demonstrate herein that the relative abundance/distribution of T_reg_ and NKT cells in VAT from Ob and CRC-affected subjects correlates with the expression of the ω6 PUFA AA and the ω3 PUFA DHA and EPA.

These results add further evidence for the presence of a regulatory/suppressive VAT microenvironment in obesity and CRC, highlighted by our previous demonstration of an increased IL-10 production, reduced immune-stimulatory properties of DC, and impaired generation of γδ T cell-mediated responses induced *ex vivo* by adipocyte microenvironment (11). In this context, the composition of VAT, in particular in FA that reflects their dietary intake, may represent an important determinant in shaping the immune cell phenotype and in influencing processes/events occurring in distal tissues that may set the basis for tumor establishment. A deep characterization of the local and systemic immune profile, allowing to distinguish between the subsets that drive pro-tumorigenic inflammation or control it, is a key issue for the comprehension of the mechanisms involved in the establishment of a tumor-favorable environment in obesity, and for the definition of more effective cancer prevention strategies based on dietary interventions.

## Ethics Statement

Investigation has been conducted in accordance with the ethical standards and with the Declaration of Helsinki, and according to national and international guidelines. It was approved by the institutional review board of Istituto Superiore di Sanità. All enrolled subjects were provided with complete information about the study and asked to sign an informed consent.

## Author Contributions

GD, MC, MP, BV, and LC designed and performed experiments and analyzed data for the characterization of PB immune cells. BS, RV, MD, and RM contributed to VAT sample collection, processing, and metabolic evaluation. IP, SP, and VB designed and performed experiments and analyzed data for the characterization of VAT SVF immune cells. LC, SG, and MC provided important contribution to the conception of the work as well as interpretation of data and manuscript writing. MP, RM, and SP provided intellectual input throughout the study.

## Conflict of Interest Statement

The research was conducted in the absence of any commercial or financial relationships that could be construed as a potential conflict of interest.
