# Supplementary material for: Distinct Blood and Visceral Adipose Tissue Regulatory T Cell and Innate Lymphocyte Profiles Characterize Obesity and Colorectal Cancer
Source: Front Immunol. 2017 Jun 9;8:643. doi: 10.3389/fimmu.2017.00643 (PMC5465245; doi:10.3389/fimmu.2017.00643)
Supplement: Supplementary file 1 [file Image_1.pdf]

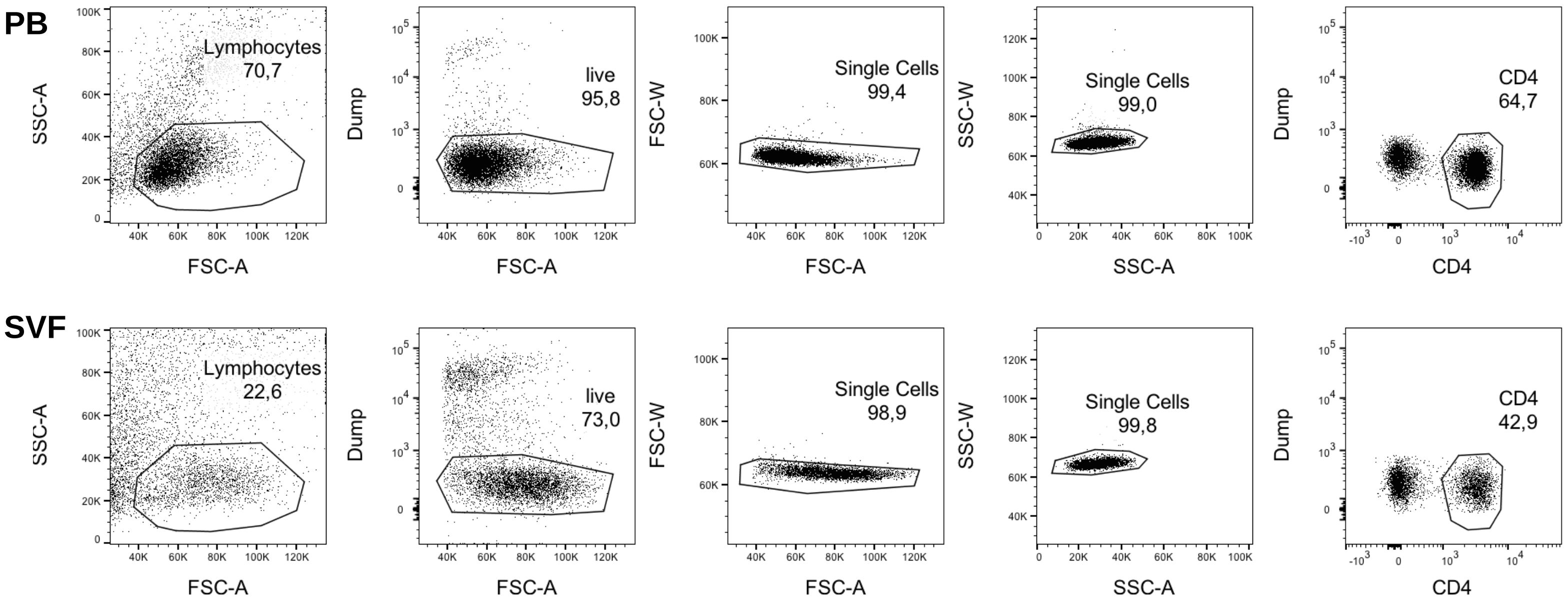

Figure S1. Gating strategy. Identification of live single lymphocytes, and in particular CD4$^+$ T cells, in PB and SVF from a representative healthy lean subject.
