# Supplementary material for: Distinct Blood and Visceral Adipose Tissue Regulatory T Cell and Innate Lymphocyte Profiles Characterize Obesity and Colorectal Cancer
Source: Front Immunol. 2017 Jun 9;8:643. doi: 10.3389/fimmu.2017.00643 (PMC5465245; doi:10.3389/fimmu.2017.00643)
Supplement: Supplementary file 2 [file Image_2.pdf]

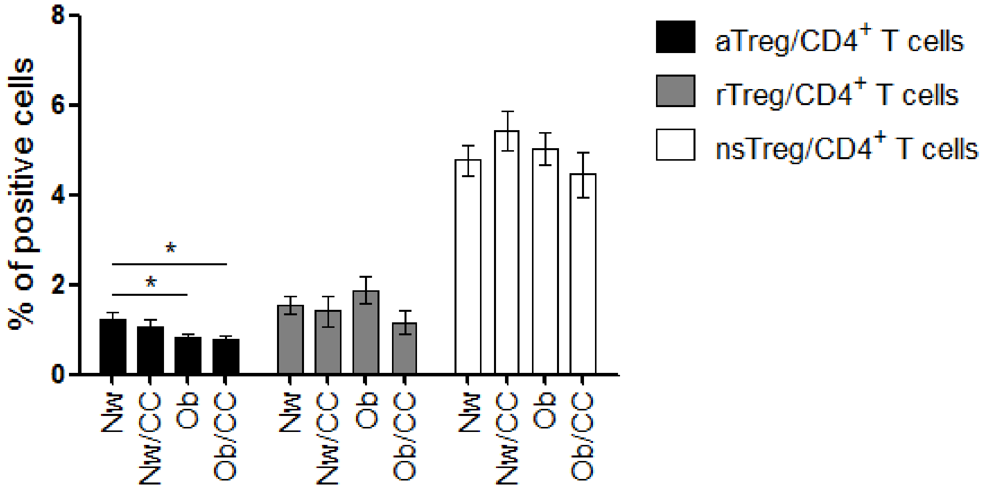

Figure S2. T_{reg} cell subset frequency in PB

PB lymphocytes isolated from lean (Nw), obese (Ob), lean affected by CRC (Nw/CC) and obese affected by CRC (Ob/CC) donors were analyzed by flow cytometry. Frequencies of aT_{reg} cells, ($CD4^{+}FOXP3^{high}CD45RA^{-}$), rT_{reg} cells ($CD4^{+}FOXP3^{low}CD45RA^{+}$) and nsT_{reg} cells, ($CD4^{+}FOXP3^{low}CD45RA^{-}$) were estimated among $CD4^{+}$ T cells. Data are expressed as mean ± SEM. $^{*}p < 0.05$ by ANOVA.
