# Supplementary material for: Distinct Blood and Visceral Adipose Tissue Regulatory T Cell and Innate Lymphocyte Profiles Characterize Obesity and Colorectal Cancer
Source: Front Immunol. 2017 Jun 9;8:643. doi: 10.3389/fimmu.2017.00643 (PMC5465245; doi:10.3389/fimmu.2017.00643)
Supplement: Supplementary file 3 [file Image_3.pdf]

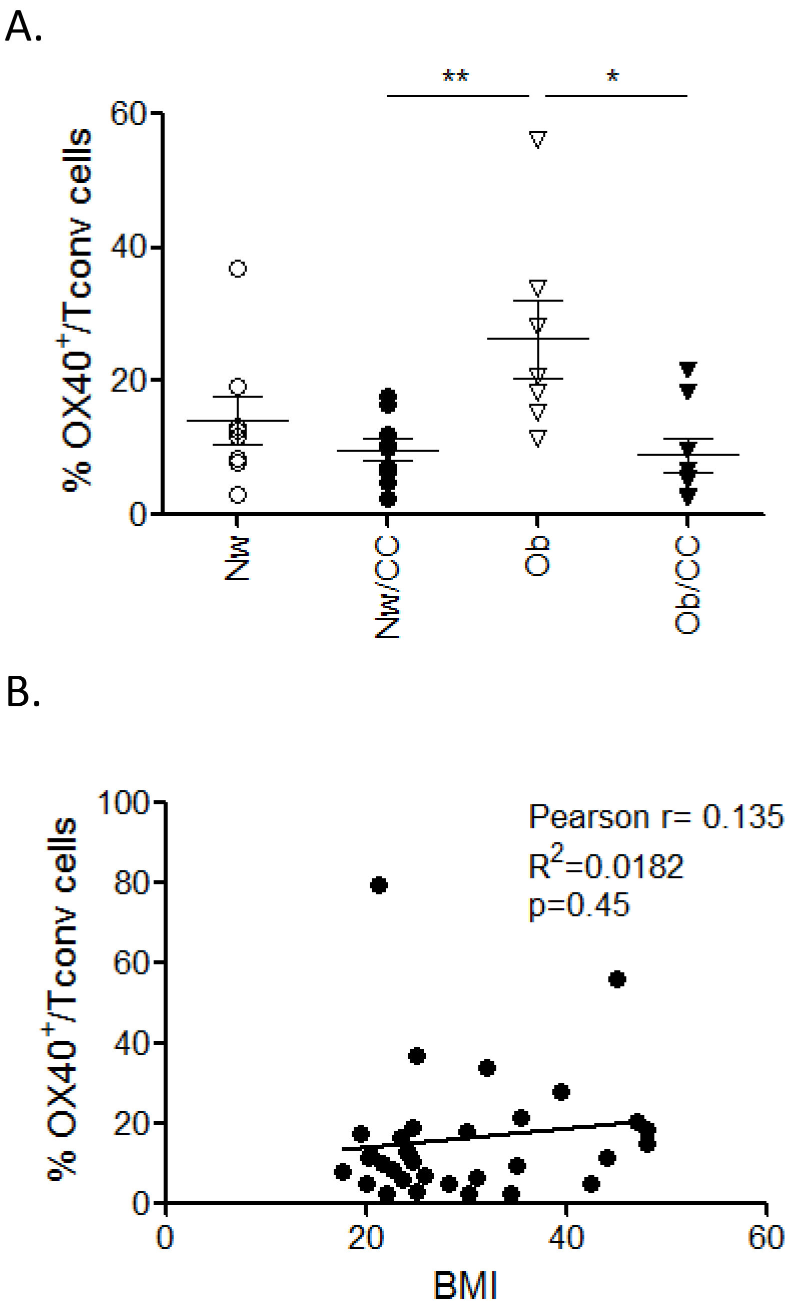

Figure S3. OX40 expression in VAT T_{conv} cells does not correlate with BMI

PB and VAT SVF lymphocytes isolated from lean (Nw), obese (Ob), lean affected by CRC (Nw/CC) and obese affected by CRC (Ob/CC) donors were analyzed by flow cytometry. Frequency of $OX40^+$ T cells (among $CD4^+FOXP3^-$ T_{conv} cells) was estimated in VAT SVF of the 4 groups (**A**). Each dot represents an individual donor. Mean ± SEM is shown for each group. $*p < 0.05$; $**p < 0.01$ by ANOVA. (**B**) Pearson's correlation (r) between $OX40^+$ T_{conv} cell frequency and BMI in all subjects.
